# Fabrication of Crack-Free Barium Titanate Thin Film with High Dielectric Constant Using Sub-Micrometric Scale Layer-by-Layer E-Jet Deposition

**DOI:** 10.3390/ma9010061

**Published:** 2016-01-19

**Authors:** Junsheng Liang, Pengfei Li, Dazhi Wang, Xu Fang, Jiahong Ding, Junxiong Wu, Chang Tang

**Affiliations:** Key Laboratory for Micro/Nano Technology and System of Liaoning Province, Dalian University of Technology, Dalian 116023, China; lipengfeizuoyou@mail.dlut.edu.cn (P.L.); d.wang@dlut.edu.cn (D.W.); prancer@mail.dlut.edu.cn (X.F.); dingjiahong@mail.dlut.edu.cn (J.D.); wujunxiong@mail.dlut.edu.cn (J.W.); tangchang@mail.dlut.edu.cn (C.T.)

**Keywords:** BTO, Thin film, Crack-free, E-jet deposition

## Abstract

Dense and crack-free barium titanate (BaTiO_3_, BTO) thin films with a thickness of less than 4 μm were prepared by using sub-micrometric scale, layer-by-layer electrohydrodynamic jet (E-jet) deposition of the suspension ink which is composed of BTO nanopowder and BTO sol. Impacts of the jet height and line-to-line pitch of the deposition on the micro-structure of BTO thin films were investigated. Results show that crack-free BTO thin films can be prepared with 4 mm jet height and 300 μm line-to-line pitch in this work. Dielectric constant of the prepared BTO thin film was recorded as high as 2940 at 1 kHz at room temperature. Meanwhile, low dissipation factor of the BTO thin film of about 8.6% at 1 kHz was also obtained. The layer-by-layer E-jet deposition technique developed in this work has been proved to be a cost-effective, flexible and easy to control approach for the preparation of high-quality solid thin film.

## 1. Introduction

Recently, barium titanate (BaTiO_3_, BTO) thin film has attracted potential interest in many fields such as inorganic-organic nanocomposites [1], humidity sensors [2], piezoelectric and positive temperature coefficient devices [3], ferroelectric memories [4] and integrated on-chip capacitors (ICOs) [5]. However, the crack and porosity control is still a challenge for the preparation of BTO thin films when made using traditional techniques such as hydro-thermal synthesis [6] and electrophoretic deposition [7]. The pores and cracks in BTO thin film will result in low dielectric constant and high dissipation factor [3,8].

In order to tackle this challenge, great efforts have been made to control the deposition, drying and sintering parameters in BTO thin film preparations. Ashiri *et al.* [9] had successfully developed a cost-effective technique for the preparation of tens-of-nanometers-thick BTO thin film via sol-gel dip-coating. Dense and crack-free BTO thin film was obtained from this method by finely controlling the solvent evaporation and pre-drying process. On the other hand, high-density nanostructured BTO ceramic was synthesized by Alves and co-workers [10] using an innovative processing route which involves high-energy ball-milling and spark plasma sintering. The as-prepared dense BTO thin film with high dielectric permittivity has shown potential application in multilayer capacitors. The above-mentioned processing techniques had been proved to be ideal methods to obtain dense and crack-free BTO thin films. However, the extensive use of these methods was still limited by their rather complex process steps or equipment needs.

In this work, a novel technique for BTO thin film preparation using sub-micrometric scale layer-by-layer electrohydrodynamic jet (E-jet) deposition was developed. The E-jet deposition makes use of electrical and mechanical forces to form a liquid jet, which will further disintegrate into droplets and finally be deposited on the receiver plate and form a continuous film [11,12]. Compared with other thin film deposition methods, such as ink jet printing [13] and dispensing [14], the E-jet technique in this work can easily obtain large area and uniform deposition of nano-scale droplets by electrostatic atomization of droplets at the end of the jet, which will enable the thin film quality of layer-by-layer deposition to be more precisely controlled. In a layer-by-layer E-jet deposition process, the thickness of each deposition layer was done on a sub-micrometric scale (less than 400 nm in this work), and cracks and pores in the former deposition layer can be filled in time by the later deposition to finally obtain dense and crack-free BTO thin film. Furthermore, influences of the deposition parameters, including jet height and line-to-line pitch, on the micro-structure of the BTO thin film were also studied. 

## 2. Experimental Details

### 2.1. Preparation of the BTO Slurry

The electrohydrodynamic process can form different jet modes according to different suspension properties such as viscosity, conductivity and surface tension under a certain electric field [15]. In these jet modes, only the stable cone-jet mode can be applied to the E-jet deposition. Therefore, physical properties of the suspension ink containing BTO nanopowder (with average diameter of 30 nm) and BTO sol from sol-gel processing need to be adjusted to a rational range before it can be used in the E-jet deposition. 

To prepare the BTO sol, 5.105 g of tetrabutyl titanate (99.9 wt. % purity) and equimolar barium acetate (99.9 wt. % purity) were dissolved in 2 mL of 1-propylalcohol (99.9 wt. % purity), then 20.5 mL of glacial acetic acid (99.9 wt. % purity) were also added. After being stirred for 30 min in planetary ball mill at the speed of 300 rpm, the precursor solution was hydrolyzed by adding 1.62 g of deionized water. The final liquid was further stirred at the speed of 1000 rpm until the precipitate completely disappeared and colorless, radiation-transparent and stable BTO sol was obtained. The whole process was operated in the dry N_2_ atmosphere.

**Figure 1:**
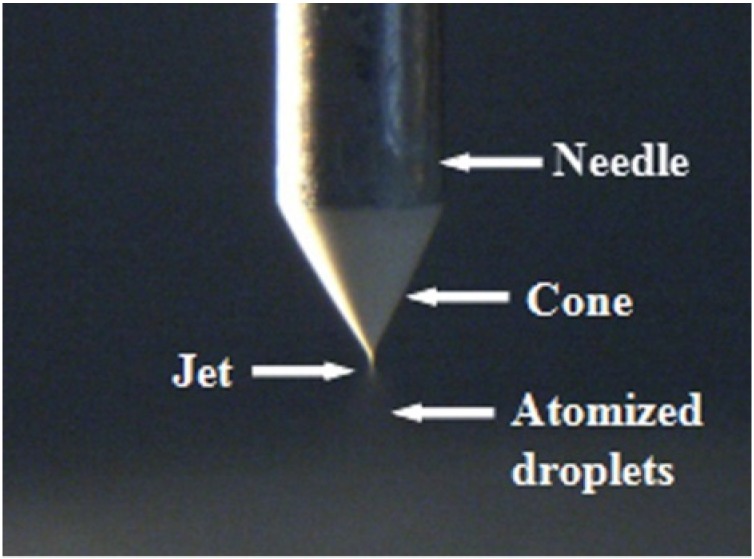
Cone-jet mode of the E-jet deposition.

The composition of the BTO slurry was 8.5 g of BTO powder, 10 mL of BTO sol, 0.4 g of dispersant (KR55, Kenrich Petrochemicals, Bayonne, NJ, USA) and 100 g of zirconia ball-milling media. The slurry was ball-milled for 48 h to maintain its stability and make it suitable to form a cone-jet mode under the combined effect of the fluid and electric field (Figure 1). For the dissolvent loss compensation during the ball-milling, 2.2 mL 1-propylalcohol and 2.0 mL glacial acetic acid were added. The property of the BTO slurry finally obtained in this work is listed in Table 1.

**Table 1:** Properties of the BTO slurry.

Properties	Density ρ/(kg·m^−3^)	Viscosity µ/(mPa·s)	Surface Tension σ/(N·m^−1^)	Conductivity K/(S·m^−1^)
BTO slurry	1.53 × 10^3^	6.980	2.539 × 10^−2^	3.794 × 10^−2^

### 2.2. Thin Film Deposition

In this experiment, the thickness of a single BTO deposition layer is about 400 nm, and two-layer deposition by the cross-scan makes up one deposition cycle. To prevent cracks, the BTO thin film was successively pre-dried at 150 °C for 120 s and 350 °C for 120 s after each deposition cycle to remove all the organic components. The pre-dried film was then sintered at 900 °C for 2 h in a muffle furnace to form perovskite structure. 

**Figure 2:**
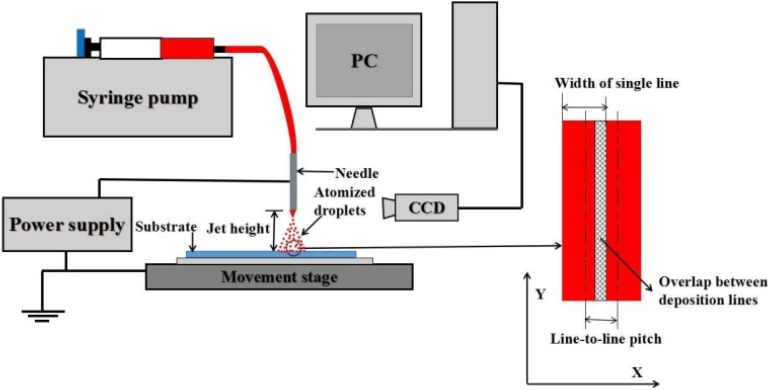
Schematic of the E-jet deposition apparatus.

A home-made E-jet deposition apparatus as schematically shown in Figure 2 was used in this work. It mainly comprises a computer-controlled X-Y movement stage, a high-voltage DC power supply and a syringe pump used to supply the ink. The receiver electrode for E-jet deposition was a Si substrate coated with Pt/Ti/SiO_2_ layers, which need to be in turn cleaned in acetone, ethanol and deionized water in an ultrasonic bath before deposition. To evaluate the dielectric properties of the thin film, 200-nm-thick copper electrode with a diameter of 3.7 mm was deposited on the sintered thin film by vacuum evaporation.

During the E-jet deposition, working distance between the jet needle and the ground electrode (the so-called jet height) need to be elaborately selected. The scan speed for a single layer deposition was set to 50 mm/s, and the ink flow rate was fixed to 4 μL/min. The total area of the deposited thin film was 20 mm × 20 mm, which can be completely covered by equally spaced parallel deposition lines. The interval between two neighboring deposition lines (the line-to-line pitch) was set to 200–500 μm to ensure a proper overlap. A single layer deposition can be completed within 40 to 18 s depending on different line-to-line pitches. The microstructure of the deposition BTO thin film was observed by emission scanning electron microscopy (SEM, JEOL JSM-6360LV, Tokyo, Japan). Energy-dispersive X-ray diffraction spectroscope (XRD-6000, Shimadzu Corp, Kyoto, Japan) was used to study the crystal orientation of the BTO thin film. The dielectric properties were characterized by an impedance analyzer.

## 3. Results and Discussion

### 3.1. Influence of Jet Height

**Figure 3:**
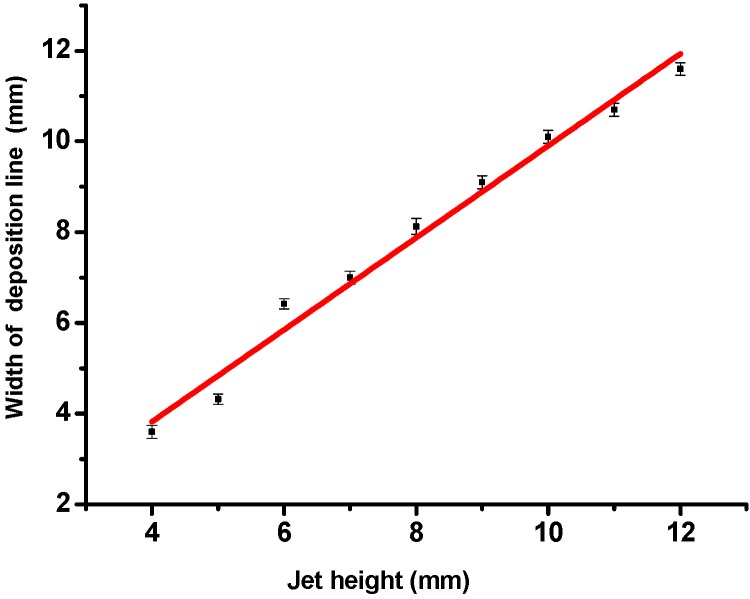
Relationship between jet height and width of a single deposition line.

The jet height is an important factor which not only influences the E-jet mode, but also directly affects the width of a single deposition line, which is defined as the spreading width of the atomization droplets along the scanning direction of the jet needle. The relationship between the jet height and width of a single deposition line on the substrate is shown in Figure 3. It is clear that the line width increases linearly with the increase of the jet height, because the atomization area of the E-jet has an inverted-cone shape. With the increment of the cone height, the diameter of the atomization cross-section, namely the line width of the deposition, will be proportionally increased.

**Figure 4:**
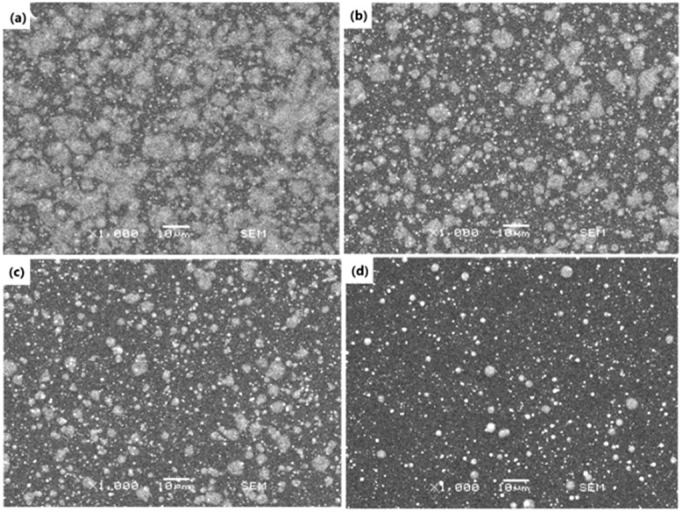
SEM images of the BTO single layer fabricated with different jet heights: (**a**) 4 mm; (**b**) 5 mm, (**c**) 6 mm and (**d**) 10 mm.

**Figure 5:**
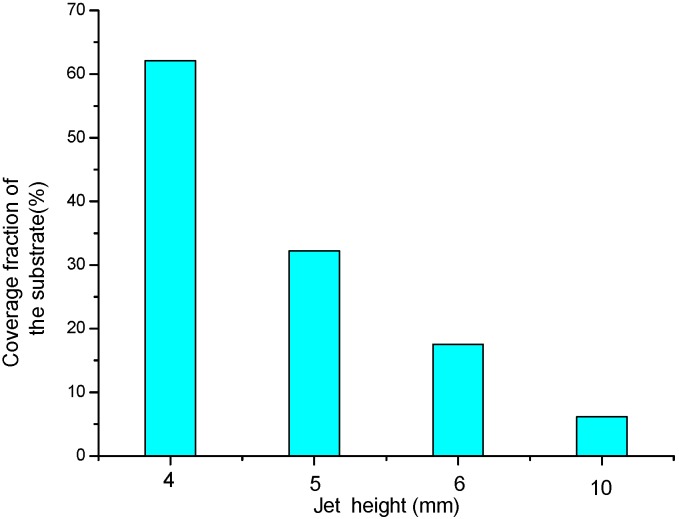
Relationship between jet height and coverage fraction of the substrate.

Figure 4 shows the top-view SEM images of the BTO single layer obtained at different jet heights. In this figure, the cloud-shaped deposition results from the sintered slurry, and the particulates are from the BTO powder agglomeration. These agglomerations were caused by the incomplete coverage of the E-jet droplets on the substrate. To evaluate the droplets’ coverage at different jet heights, the coverage fraction, which is defined as the ratio of coverage area to total area of the deposited film, is introduced. In this work, the sum coverage area of the droplets can be obtained from the SEM images in Figure 4 by using pixels’ gray-level threshold statistics and contour extraction algorithm. It is clear that the coverage fraction of the BTO droplets on the Si substrate declines with the increment of the jet height. Figure 5 gives a quantitative result of the coverage fraction at different jet height, which shows a sharp decrease in an exponential form from 62.1% at a jet height of 4 mm to only 6.16% at 10 mm. This is because, when the jet height is increased, the E-jet droplets will undergo a more adequate split and atomization process during their falling, become smaller and be dispersed on a larger area on the substrate. A too-low coverage fraction of droplets will reduce the continuity of the single deposition layer and increase the difficulty of defect filling by the later deposition layers, which may correspondingly increase the porosity and crack risk of the thin films. This will be observed in the subsequent deposition of multilayer thin films.

**Figure 6:**
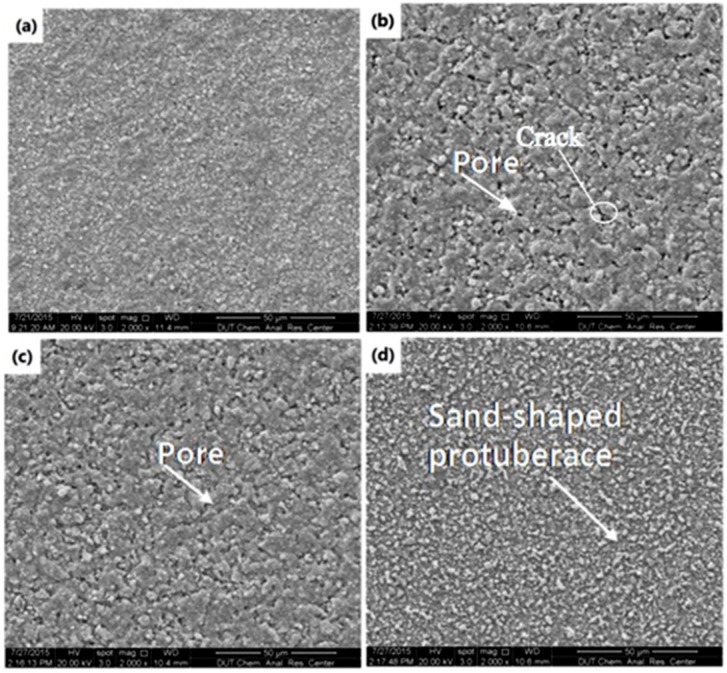
SEM images for four-layer BTO thin film deposited with different jet heights: (**a**) 4 mm; (**b**) 5 mm, (**c**) 6 mm and (**d**) 10 mm.

The micro-structure of the thin film surface is also impacted by the jet height, as illustrated in Figure 6, which shows the top-view SEM images of four-layer BTO thin films prepared with different jet heights. It is clear that the BTO thin film fabricated with a 4 mm jet height is smooth and no cracks appear on its surface (Figure 6a). However, with the increase of the jet height, cracks and pores on the film are found (Figure 6b,c). At a 10 mm jet height, the surface becomes rougher and is bestrewn with sand-shaped protuberances (Figure 6d). This means that smaller jet height is beneficial to the film deposition because larger E-jet slurry droplets generated at a small jet height can repair the deficient film formed in the former layer deposition more efficiently.

### 3.2. Impacts of Line-to-Line Pitch

**Figure 7:**
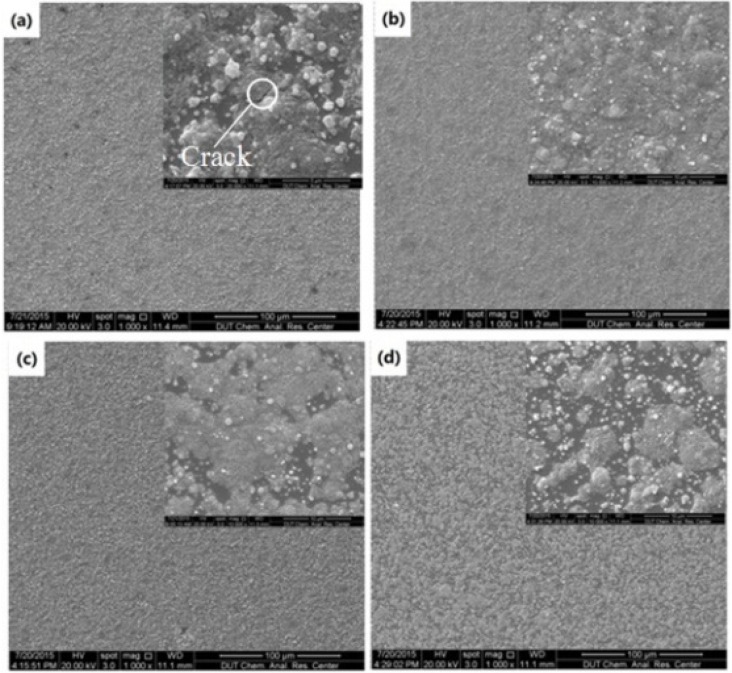
SEM images for two-layer BTO thin film at 4 mm jet height with different line-to-line pitches: (**a**) 200 μm; (**b**) 300 μm; (**c**) 400 μm and (**d**) 500 μm.

In this work, a single layer of BTO thin film was formed by splicing the parallel deposition lines, so the overlap between deposition lines also has great impact on the film structure. At a 4 mm jet height, as shown in Figure 7a, when the line-to-line pitch was set to 200 μm, cracks were observed on the film. This is because the smaller pitch will produce a thicker deposition layer, and the stress induced by solvent evaporation and volume shrinkage of the sol will be more difficult to release in time. After the pitch was increased to 300 μm, a crack-free and smooth deposition layer was obtained (Figure 7b). It means that this pitch value can well balance the coverage fraction and inner stress of the deposition layer. However, when the pitch was further increased to 400 and 500 μm, the substrate was not completely covered (Figure 7c,d). This will result in a too-rough deposition layer and higher porosity of the BTO thin film.

### 3.3. Thin Film Deposition

**Figure 8:**
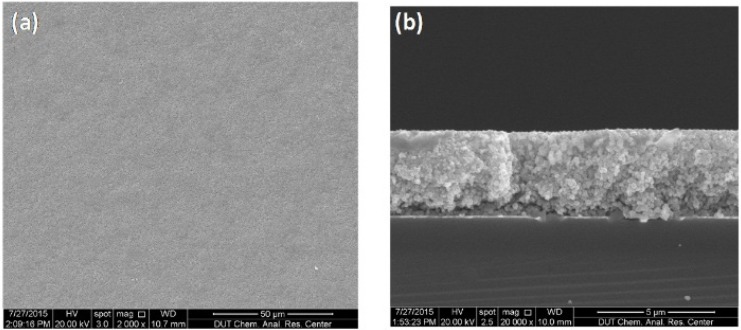
SEM images for 10-layer BTO thin film prepared with 4 mm jet height and 300 μm line-to-line pitch: (**a**) top-view; (**b**) cross-section view.

**Figure 9:**
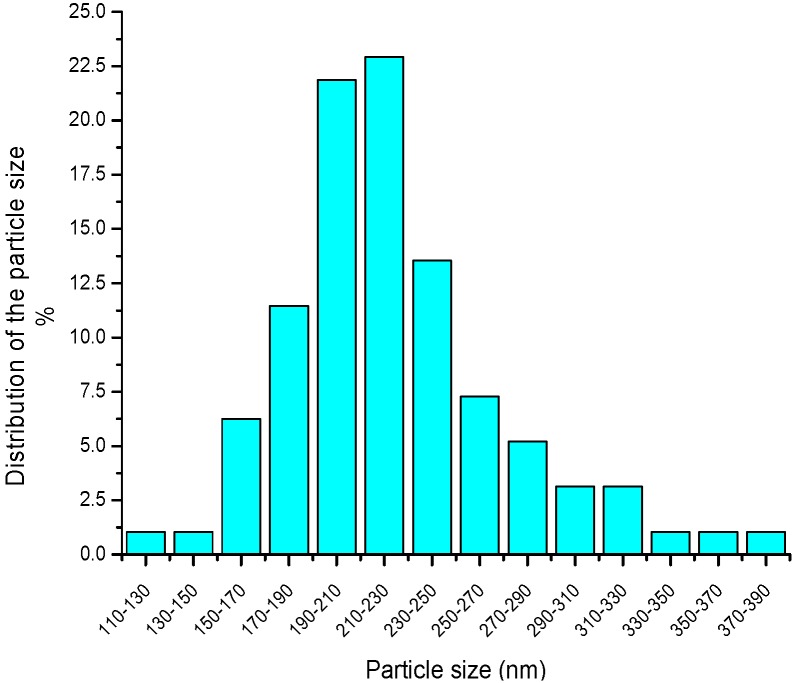
The distribution of the particle size on the cross-section.

**Figure 10:**
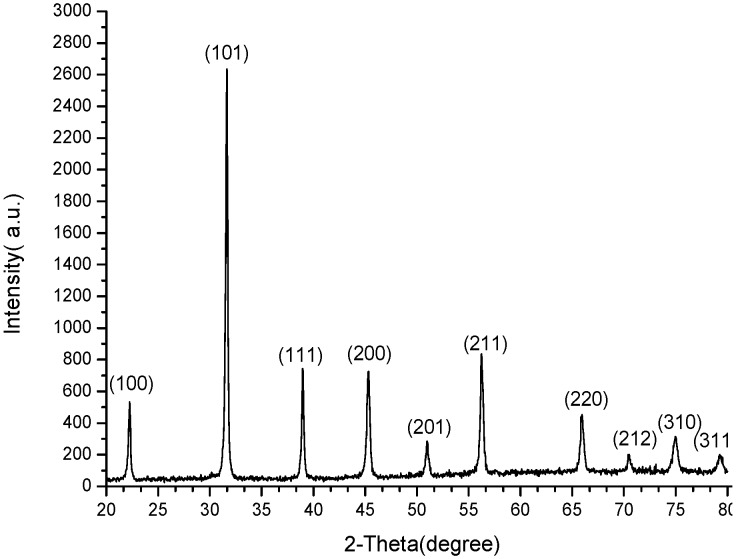
XRD pattern of the BTO film sintered at 900 °C for 2 h.

By using proper deposition parameters (4 mm jet height and 300 μm line-to-line pitch), a 3.7 μm thick, 10-layer BTO thin film (average thickness of each deposition layer is 370 nm) was deposited on the Si substrate and then sintered at 900 °C for 2 h. The top-view and cross-section SEM images of the thin film are respectively shown in Figure 8a,b. It is found that the deposited thin film is smooth and crack-free. The average grain size was also measured as about 190~220 nm from Figure 8b by using the software ImageJ©, as illustrated in Figure 9. The XRD pattern in Figure 10 shows that only perovskite structure was formed in the BTO thin film after sintering. This means that the BTO nano-powder and sol in the deposited film can be fully converted to a crystallized cubic perovskite structure at a sintering temperature higher than 900 °C, as also has been reported in the previous literature [4,16].

### 3.4. Dielectric Properties

**Figure 11:**
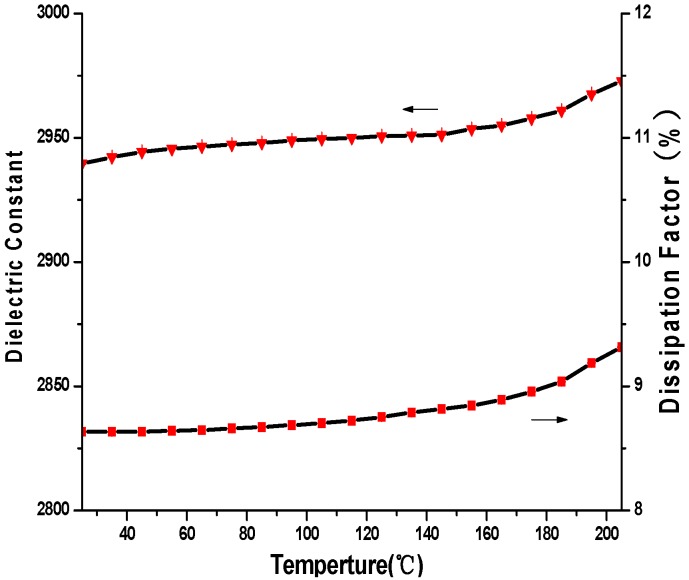
Temperature dependence of dielectric constant and dissipation factor of the BTO thin film at 1 kHz.

**Figure 12:**
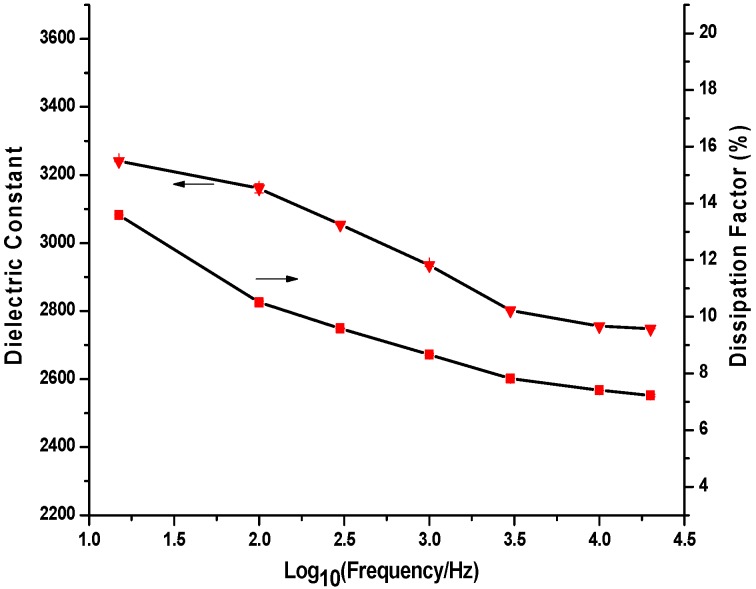
Frequency dependence of dielectric constant and dissipation factor for the BTO thin film at 25 °C.

Figure 11 shows the temperature dependence of the dielectric constant and the dissipation factor of the as-prepared BTO thin film measured at 1 kHz. It can be seen that the dielectric constant and dissipation factor were separately measured as 2940 and 8.6% at 25 °C, and both have a slow increase as the temperature rises from 25 °C to 205 °C. At 205 °C, the dielectric constant and dissipation factor were recorded as 2970 and 9.3%, respectively. However, no dielectric peak was found at the Curie temperature point, which is 130 °C for the BTO thin film [17]. It means that there is no phase transition from tetragonal to cubic in the BTO thin film at its Curie temperature point in this study. This phenomenon also had been reported by Lee and co-authors in their previous research work [17]. Figure 12 shows the frequency dependence of the dielectric constant and dissipation factors at room temperature (25 °C). It is clear that both the dielectric constant and dissipation factor decrease with the increase of the measure frequency. When the frequency was increased from 15 Hz to 3 kHz, the dielectric constant of the BTO thin film dropped from 3241 to 2801, and then slowly approached to about 2748 with the further increase of the frequency to 20 kHz. Meanwhile, the dissipation factor of the BTO thin film will also decline from 13.6% at 15 Hz and 8.6% at 1 kHz, to 7.8% at 3 kHz, and then gradually approach to about 7.2% at 20 kHz.

## 4. Conclusions

By using sub-micrometric scale, layer-by-layer E-jet deposition technique, crack-free and dense BTO thin films were successfully prepared on the Si substrate. Experimental results show that the jet height and line-to-line pitch play important roles for the E-jet deposition of the film. Crack-free and homogeneous BTO thin film structure can be obtained by using a 4 mm jet height and 300 μm line-to-line pitch in this work. The dielectric constant of the prepared BTO thin film was recorded as high as 3241 at 15 Hz and 2940 at 1 kHz at room temperature. Meanwhile, the low dissipation factor of the BTO thin film of about 8.6% at 1 kHz and 7.2% at 20 kHz was also obtained. The dielectric properties of the BTO thin film can be further improved by optimizing the deposition parameters in the future work. Additionally, the layer-by-layer E-jet deposition technique applied in this work has been proved to be an effective, flexible and easy-to-control method for the preparation of high-quality, solid thin film.
